# Meta-Analysis of the Prevalence of Porcine Zoonotic Bacterial Pathogens in India: A 13-Year (2010–2023) Study

**DOI:** 10.3390/pathogens12101266

**Published:** 2023-10-21

**Authors:** Swaraj Rajkhowa, Joyshikh Sonowal, Udipta Borthakur, Seema Rani Pegu, Rajib Deb, Pranab Jyoti Das, Gyanendra Singh Sengar, Vivek Kumar Gupta

**Affiliations:** 1 ICAR-National Research Centre on Pig, Rani, Guwahati 781131, Assam, India; joyshikh@gmail.com (J.S.); drseemapegu@yahoo.com (S.R.P.); drrajibdeb@gmail.com (R.D.); drpranabjotidas@gmail.com (P.J.D.); gsengar71@gmail.com (G.S.S.); gupta.drvivek@gmail.com (V.K.G.); 2 Animal Husbandry and Veterinary Department, Guwahati 781003, Assam, India; udborthakur@gmail.com

**Keywords:** India, meta-analysis, pig, prevalence, zoonotic bacteria

## Abstract

The presence of bacterial pathogens such as *Brucella* spp., *Clostridium* spp., *E. coli*, *Listeria monocytogenes*, *Salmonella* spp., *Staphylococcus* spp., and *Streptococcus suis* not only hampers pig production but also carries significant zoonotic implications. The present study aims to conduct a comprehensive meta-analysis spanning over 13 years (2010–2023) to ascertain the prevalence of these zoonotic bacterial pathogens in Indian pig populations. The study seeks to synthesize data from diverse geographic regions within India and underscores the relevance of the One Health framework. A systematic search of electronic databases was meticulously performed. Inclusion criteria encompassed studies detailing zoonotic bacterial pathogen prevalence in pigs within India during the specified timeframe. Pertinent information including authors, publication year, geographical location, sampling techniques, sample sizes, and pathogen-positive case counts were meticulously extracted. The meta-analysis of zoonotic bacterial pathogens in Indian pig populations (2010–2023) unveiled varying prevalence rates: 9% *Brucella* spp., 22% *Clostridium* spp., 19% *E. coli*, 12% *Listeria monocytogenes*, 10% *Salmonella* spp. and *Streptococcus suis*, and 24% *Staphylococcus* spp. The application of random effects further revealed additional variability: 6% *Brucella* spp., 23% *Clostridium* spp., 24% *E. coli*, 14% *Listeria monocytogenes*, 10% *Salmonella* spp. and *Streptococcus suis*, and 35% *Staphylococcus* spp. Notably, the observed heterogeneity (I^2^) varied significantly from 87% to 99%. The meta-analysis findings underscore the pervasive nature of these diseases throughout India’s pig populations, accentuating the substantial impact of these pathogens on pig health and the potential for zoonotic transmission. The present study reinforces the importance of the adoption of a comprehensive One Health approach that acknowledges the intricate interplay between animal, human and environmental health.

## 1. Introduction

The prevalence of zoonotic bacterial pathogens in animal populations poses substantial challenges to both animal and human health, calling for comprehensive assessments to inform effective management strategies. It is of particular concern for a country like India where pig husbandry plays a pivotal role in uplifting the socio-economic status of the people, especially the tribal masses for whom pig rearing is a way of life. Pork is a high-risk source of foodborne diseases worldwide. Zoonotic bacterial pathogens, such as *Brucella* spp., *Clostridium* spp., *E. coli*, *Listeria monocytogenes*, *Salmonella* spp., *Staphylococcus* spp., *Mycobacterium* spp., *Campylobacter* spp., and *Streptococcus suis* have been identified as detrimental to both pig health and public health due to their potential for zoonotic transmission. Individuals closely involved in pig farming, including pig handlers and those who consume pork products under unhygienic conditions, are highly susceptible to infections by these zoonotic bacterial pathogens. These pathogens utilize a range of mechanisms to cause diseases, such as releasing toxins, possessing virulence factors, evading the host’s immune system and establishing chronic infections within the host. In clostridial infection, *Clostridium perfringens* releases alpha toxin [1], while *Clostridium difficile* produces toxins A (TcdA, enterotoxin A) and B (TcdB, cytotoxin B), which target the colon’s lining, causing colitis and severe diarrhoea [2]. Enterohemorrhagic *E. coli* (EHEC) produces Shiga toxins, specifically Shiga toxin 1 (Stx1) and Shiga toxin 2 (Stx2), leading to severe foodborne illnesses [3], while Extended Spectrum Beta-Lactamase (ESBL) *E. coli* resists many antibiotics due to enzyme production, posing treatment challenges [4,5]. *Listeria monocytogenes* produces listeriolysin O, causing listeriosis [6]. *Salmonella* spp. exhibit resilience to gastric acidity, enabling colonization of the gastrointestinal tract and subsequent invasion of the intestinal mucosa; they produce endotoxins, such as lipid A which can trigger inflammatory responses, and exotoxins, including cytotoxins and enterotoxins like stn, which can damage host cells, disrupt intestinal function, and stimulate cytokine release, contributing to gastrointestinal infections [7]. While *Staphylococcus* spp. are known to produce a wide range of toxins, including staphylococcal enterotoxins, Toxic Shock Syndrome Toxin-1 (TSST-1), exfoliative toxins (ETA and ETB), haemolysins (alpha, beta, and delta), Panton-Valentine Leukocidin (PVL), and superantigens (such as TSST-1 and various staphylococcal enterotoxins) [8,9]. The pathogenicity of Staphylococcal toxins is associated with various clinical conditions, from food poisoning to severe skin and systemic infections. *Streptococcus suis* produces a range of virulence factors, including extracellular enzymes for tissue damage and immune evasion, adhesins for host cell attachment and streptolysins, like suilysin, which induce cell lysis and tissue damage, collectively enhancing its pathogenicity [10,11]. *Streptococcus pyogenes*, on the other hand, produces toxins like streptolysins (SLO, SLG, and SLS haemolysins), pyrogenic exotoxins, streptococcal superantigens (SAgs), streptokinase, and hyaluronidase, which collectively contribute to tissue damage, immune system overstimulation, and clinical symptoms like strep throat and necrotizing fasciitis [12,13,14]. In contrast, *Brucella* spp. primarily cause brucellosis with toxin production playing a minor role [15]. The major virulence factors of *Brucella* are lipopolysaccharide (LPS), the Type IV Secretion System (T4SS), and the BvrR/BvrS system, to interact with host cells, create specialized vacuoles (Brucella Containing Vacuole (BVC)), and establish connections with the endoplasmic reticulum, enhancing their ability to cause chronic infection within host cells [16,17].

It is known that almost two-thirds of the pathogens that cause diseases in humans are of animal origin. Brucellosis is one of the most common, widespread zoonoses throughout the world, mainly caused by *Brucella abortus*, *Brucella melitensis* or *Brucella suis* and is transmitted to people from various animal species [18]. All Shiga-toxin-producing *E. coli* (STEC) strains are pathogenic in humans, capable of causing at least diarrhoea. Depending on the presence of certain stx subtypes and the presence/absence of the *eae* gene, all STEC subtypes may be associated with severe outcomes, i.e., haemolytic uraemic syndrome (HUS), bloody diarrhoea (BD), kidney failures, hospitalizations, and deaths [19]. Pigs are important reservoirs of STEC. The entrance of these strains into the food chain implies a risk to consumers because of the severity of haemolytic uremic syndrome [20]. *Clostridium difficile* is a well-established pathogen of both humans and animals that contaminates foods and the environment. To manage *Clostridium difficile* infections (CDI), a One Health approach with the collaboration of clinicians, veterinarians, environmentalists, and policy-makers is paramount. Listeriosis, a zoonotic disease caused by *Listeria monocytogenes*, is a major public health problem and one of the most common notifiable foodborne diseases [21]. It has also been observed that pigs are an important reservoir for *L. monocytogenes* and in particular, younger animals are at risk for asymptomatic carriage [22]. Salmonellosis is one of the most serious zoonotic diseases in the world and pigs are one of the most common sources of *Salmonella* infections in humans [23]. *Streptococcus suis* is considered one of the most important pathogens affecting pig production worldwide and is also an emerging zoonotic agent in humans [24]. Methicillin-resistant *Staphylococcus aureus* (MRSA) infections can occur in both humans and pigs, leading to a range of illnesses, from skin and soft tissue infections to more severe systemic infections [25]. ESBL *E. coli* and MRSA’s resistance to multiple antibiotics complicates treatment, and it poses a public health concern due to its potential for community- and hospital-acquired infections [26,27]. The emergence of multi-drug-resistant pathogens in pig populations, driven by genetic mutations and selective pressures from antimicrobial use, threatens both animal health and public safety. Resistant bacteria of pig origin can be transmitted to humans through direct contact, environmental contamination, and the consumption of pork and its products, raising significant concerns about the spread of antimicrobial resistance. Addressing this issue requires judicious use of antibiotics in pig farming, improved biosecurity measures, and a One Health approach that recognises the interconnectedness of animal, environmental, and human health.

In this context, conducting a meta-analysis to determine the prevalence of various zoonotic bacterial pathogens in Indian pig populations is very much essential. Meta-analysis offers a powerful approach to synthesise data from various studies, providing a comprehensive overview of the prevalence landscape. By collating and analysing prevalence data from different geographic regions within India, this study aims to establish a clear understanding of the extent of prevalence of these pathogens in the pig population. This analysis not only aids in quantifying the extent of the issue but also contributes to the identification of potential trends and patterns that can guide targeted interventions and preventive measures. By exploring the prevalence rates of *Brucella* spp., *Clostridium*, spp., *E. coli*, *Listeria monocytogenes*, *Salmonella* spp., *Staphylococcus* spp., and *Streptococcus suis* within the Indian pig population, this meta-analysis seeks to provide valuable insights into the distribution and potential impacts of these pathogens.

## 2. Materials and Methods

### 2.1. Literature Retrieval and Data Compilation

**Figure 1:**
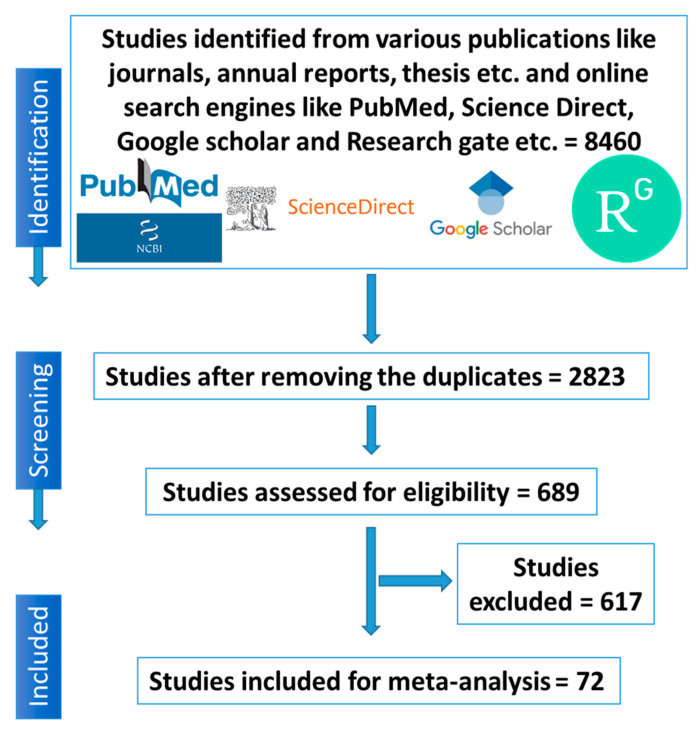
Schematic depiction of the literature selection procedure for the systematic review of the prevalence of zoonotic bacterial pathogens in swine of India from 2010 to 2023.

The process encompassed the accumulation of published studies, facilitating a methodical evaluation of the prevalence and associated risk factors of zoonotic bacterial pathogens in pigs, spanning the years 2010 to 2023. These published works were sourced from a diverse array of online search engines, such as NCBI-PubMed, Science Direct, Google Scholar, Research Gate, etc. Subsequently, an extensive review of these studies was conducted, ensuring both their quality and relevance. This review adhered to the guidelines outlined in the Preferred Reporting Items for Systematic Review and Meta-Analysis (PRISMA) and the Meta-analysis of Observational Studies in Epidemiology (MOOSE) protocols. The procedural flow is depicted in Figure 1, delineating the meticulous steps taken throughout this systematic review process.

The criteria guiding the incorporation and exclusion of studies were devised in accordance with the specifications outlined in Table 1. The relevant details within the published studies, including author details, year of publication, study location (regional designation), sample dimension, sample types, and instances of positive occurrences, were methodically extracted to facilitate the meta-analytical process. The determination of the collective prevalence of zoonotic bacterial pathogens in pigs within India was carried out distinctly for each distinct pathogen.

### 2.2. Methods Used for Meta-Analysis

**Figure 2:**
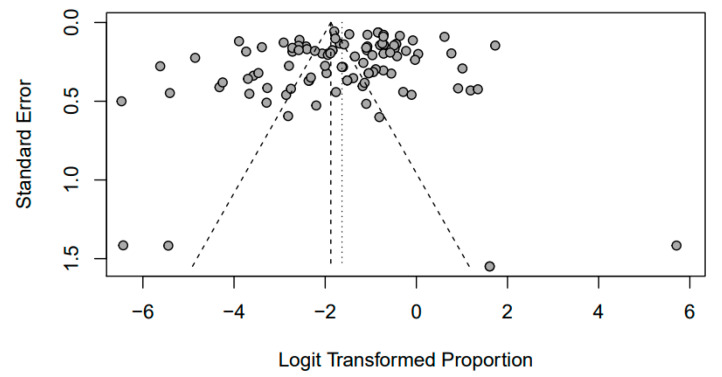
Funnel plot that elucidates potential publication bias in prevalence of zoonotic bacterial pathogens in India from 2010 to 2023.

Utilizing R-software, the prevalence of zoonotic bacterial pathogens in pigs was computed through the application of meta-analysis tools. This encompassed the systematic analysis of 73 published studies conducted across India, spanning the timeline from 2010 to 2023. A funnel plot generated using the ‘dplyr’ package in R was employed to visually assess publication bias and the potential influence of small-study effects. This plot aids in identifying any asymmetry in the distribution of effect sizes and offers insights into the presence of bias within the included studies. The presentation of a funnel plot involves the plotting of the logit proportion against the standard error. The emergence of signs suggesting publication bias implies the appropriateness of employing the random effects model for the analysis of this dataset (Figure 2).

The analysis was subdivided pathogen-wise, such as *Brucella* spp., *Clostridium* spp., *E. coli*, *Listeria monocytogenes*, *Salmonella* spp., *Staphylococcus* spp., and *Streptococcus suis* separately. The list of studies included in the meta-analysis of zoonotic bacterial pathogens in pigs is given in Table 2. The ‘meta’ package in R was employed to generate a forest plot, an effective visual tool for presenting the effect sizes and corresponding confidence intervals of individual studies. Two distinct models were employed to estimate the proportion of positive samples in relation to the sample size using Forest plots: the common effect model was used to estimate the overall prevalence of zoonotic bacterial pathogens across all studies, assuming homogeneity among the studies; the random effects model, accounting for potential heterogeneity, provided a more conservative estimate. Heterogeneity among the studies was assessed using the I^2^ statistic, which quantifies the proportion of variability attributable to true heterogeneity rather than chance. The presence of heterogeneity was considered significant at I^2^ values greater than 50%. The τ^2^ (tau-square) value was calculated to estimate the extent of true differences contributing to the observed heterogeneity.

## 3. Results

### 3.1. Meta-Analysis

**Figure 3:**
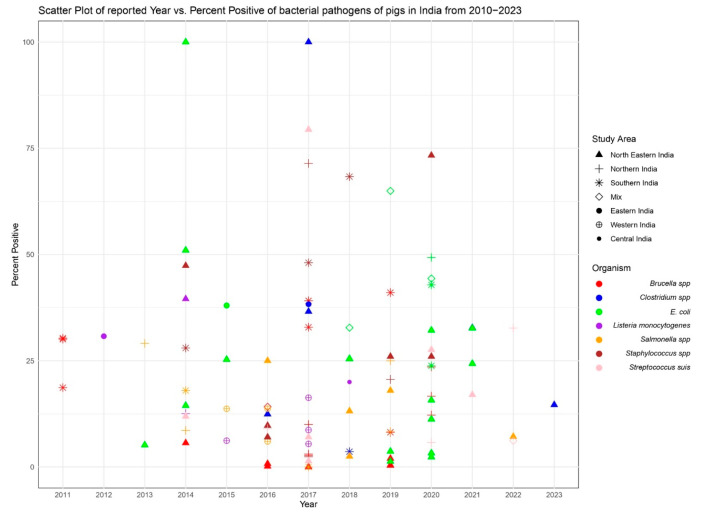
Representation of a scatter plot, depicting the positive percentage trends in the prevalence of different zoonotic bacterial pathogens among Indian swine. The data spans the years from 2010 to 2023 and includes samples from different regions across India.

The prevalence of *Brucella* spp., *Clostridium*, *E. coli*, *Listeria monocytogenes*, *Salmonella* spp., *Staphylococcus* spp., and *Streptococcus suis* were calculated separately for pigs. The meta-analysis for these organisms was carried out using 73 published studies from India, which included 23 studies on *Brucella* spp., 7 studies on *Clostridium* spp., 23 studies on *E. coli*, 8 studies on *Listeria monocytogenes*, 14 studies on *Salmonella* spp., 11 studies on *Staphylococcus* spp., and 10 studies on *Streptococcus* suis on pig from India (Figure 3).

### 3.2. Meta-Analysis of the Prevalence of Brucellosis in Pigs

**Figure 4:**
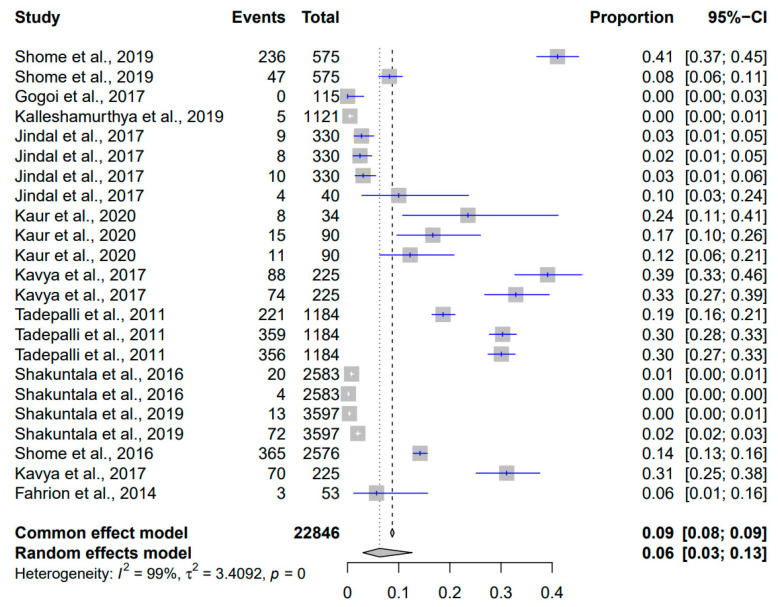
Forest plot showing the result of 23 studies reporting the prevalence of brucellosis in pigs in India from 2010 to 2023.

In this meta-analysis of the prevalence of *Brucella* spp. in pigs across India (2010–2023), a total of 22,846 events were included (Figure 4). The common effect model yielded an estimated overall prevalence proportion of nine percent (95% CI: [8%; 9%]), suggesting that approximately 9 out of every 100 pigs were infected with *Brucella* spp. in India. On the other hand, the random effects model, which accounts for potential heterogeneity among the studies, yielded an estimated proportion of six percent (95% CI: [3%; 13%]). The considerable heterogeneity observed in the random effects model, indicated by an I² value of 99%, underscores the diversity in the study outcomes beyond what could be attributed to chance. This indicates the presence of factors influencing *Brucella* prevalence differences across the studies, such as variations in sample collection methods, geographical regions, management practices and testing protocols. The associated *p*-value of zero further confirms the statistical significance of this heterogeneity. The calculated τ² value of 3.4092 highlights the extent to which true differences in *Brucella* prevalence rates among the studies contribute to the observed heterogeneity.

### 3.3. Meta-Analysis of the Prevalence of Clostridium spp. in Pigs

**Figure 5:**
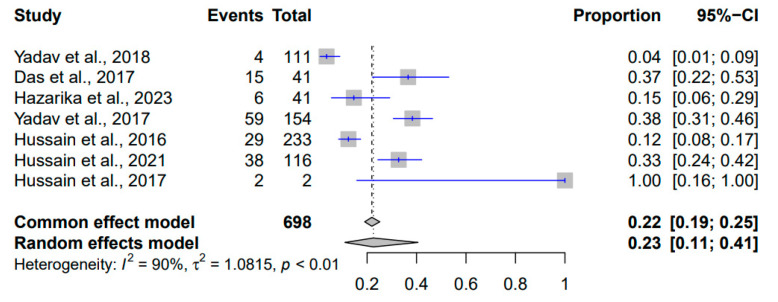
Forest plot showing the result of 7 studies reporting the prevalence of *Clostridium* spp. in pigs in India from 2010 to 2023.

The meta-analysis of the prevalence of *Clostridium* spp. in Indian pigs (2010–2023) based on 698 events revealed an estimated overall proportion of 22% (95% CI: [0.19; 0.25]) using the common effect model and 23% (95% CI: [0.11; 0.41]) using the random effects model (Figure 5). Heterogeneity was substantial (I^2^ = 90%, *p* < 0.01), suggesting diverse factors contributing to the observed variation. The τ² value of 1.0815 highlighted the degree of true differences between studies.

### 3.4. Meta-Analysis of the Prevalence of E. coli in Pigs

**Figure 6:**
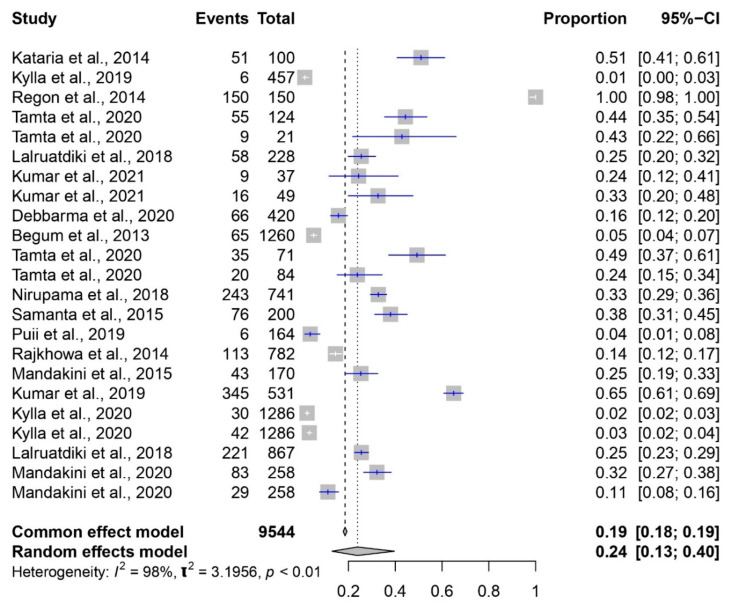
Forest plot showing the result of 23 studies reporting the prevalence of *E. coli* in pigs in India from 2010 to 2023.

In the present study, the meta-analysis of the prevalence of *E. coli* in pigs in India between 2010 and 2023 employed two distinct models to estimate the proportion of positive cases (Figure 6). The common effect model yielded an estimated prevalence of 19% (95% CI: [18%; 19%]), suggesting that about 19% of cases were associated with *E. coli* infection in the pig population during this period. The random effects model, which considers study variability, provided a slightly higher estimate of 24% (95% CI: [13%; 40%]), reflecting potential differences across studies. Heterogeneity was pronounced, with an I² value of 98%, signifying significant variation beyond chance. The τ^2^ value of 3.1956 further quantified true differences contributing to heterogeneity.

### 3.5. Meta-Analysis of the Prevalence of Listeria monocytogenes in Pigs

**Figure 7:**
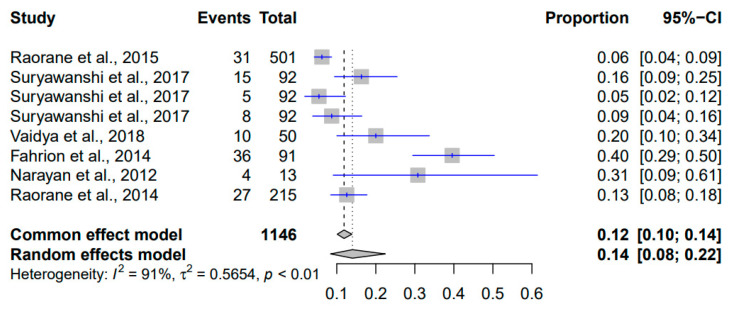
Forest plot showing the result of 8 studies reporting the prevalence of *Listeria monocytogenes* in pigs in India from 2010 to 2023.

The results of the meta-analysis of the prevalence of *Listeria monocytogenes* in Indian pigs from 2010 to 2023 are shown in Figure 7. With a total of 1146 events, the common effect model estimated a prevalence of 12% (95% CI: [10%; 14%]), suggesting that approximately 12% of pigs were affected. The random effects model estimated a prevalence of 14% (95% CI: [8%; 22%]), indicating potential study variations. Heterogeneity was significant (I^2^ = 91%), denoting substantial variation beyond chance. The *p*-value below 0.01 affirmed this heterogeneity’s statistical significance. A τ^2^ value of 0.5654 quantified genuine differences contributing to the variation.

### 3.6. Meta-Analysis of the Prevalence of Salmonella spp. in Pigs

**Figure 8:**
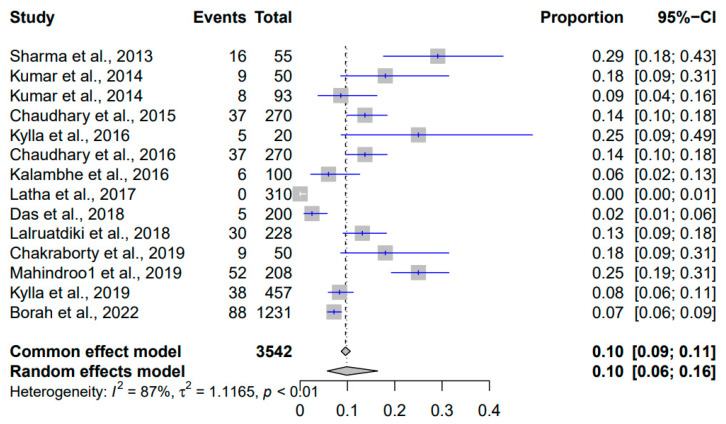
Forest plot showing the result of 14 studies reporting the prevalence of *Salmonella* spp. in pigs in India from 2010 to 2023.

The results of the meta-analysis of the prevalence of *Salmonella* spp. in Indian pigs spanning 2010 to 2023 are shown in Figure 8. With a total of 3542 events, the common effect model estimated a prevalence of ten percent (95% CI: [9%; 11%]), implying that approximately ten percent of pigs were infected. Interestingly, the random effects model produced a comparable estimate of ten percent (95% CI: [6%; 16%]), accommodating potential variations in study approaches. Substantial heterogeneity was observed (I^2^ = 87%), implying significant variation beyond chance. This heterogeneity’s statistical significance was reaffirmed by the *p*-value less than 0.01. A τ^2^ value of 1.1165 quantified the extent of authentic differences contributing to this observed variation.

### 3.7. Meta-Analysis of the Prevalence of Staphylococcus spp. in Pigs

**Figure 9:**
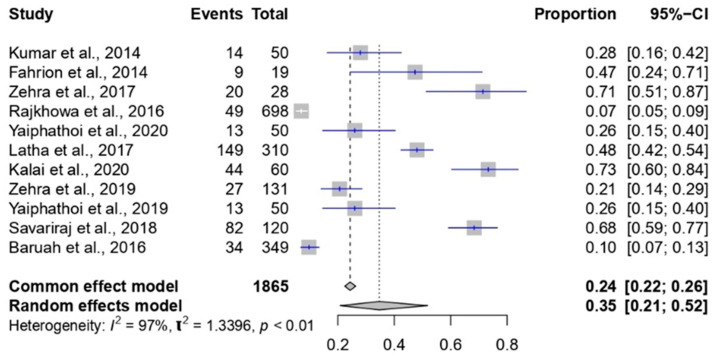
Forest plot showing the result of 11 studies reporting the prevalence of *Staphylococcus* spp. in pigs in India from 2010 to 2023.

In the current study, a meta-analysis was conducted to explore the prevalence of *Staphylococcus* spp. in Indian pigs between 2010 and 2023 (Figure 9). The dataset encompassed a total of 1865 events. The common effect model estimated a prevalence of 24% (95% CI: 22% to 26%), indicating that approximately 24% of pigs were affected by *Staphylococcus* spp. during this period. Contrastingly, the random effects model, accounting for potential study variations, presented a higher estimated prevalence of 35% (95% CI: 21% to 52%). Heterogeneity was pronounced, with an I² value of 97%, indicating substantial variation beyond chance. The associated *p*-value of less than 0.01 confirmed the statistical significance of this heterogeneity. The τ² value of 1.3396 quantified the extent to which genuine differences in *Staphylococcus* spp. prevalence rates contributed to the observed heterogeneity.

### 3.8. Meta-Analysis of the Prevalence of Streptococcus suis in Pigs

**Figure 10:**
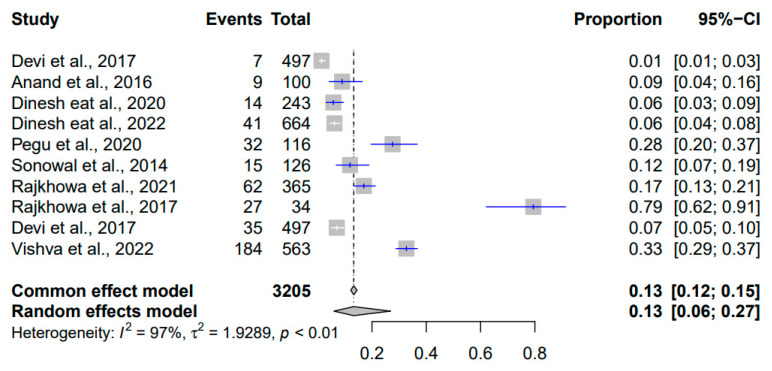
Forest plot showing the result of 10 studies reporting the prevalence of *Streptococcus suis* in pigs in India from 2010 to 2023.

The comprehensive meta-analysis investigating the prevalence of *Streptococcus suis* in Indian pigs from 2010 to 2023 analysed a total of 3205 events (Figure 10). The common effect model estimated a prevalence of 13% (95% CI: [12%; 15%]), suggesting that roughly 13% of pigs were affected by *Streptococcus suis* during this period. The random effects model, designed to account for potential variations between studies, yielded a similar estimated prevalence of 13% (95% CI: [6%; 27%]). Heterogeneity emerged with an I^2^ value of 97%, signifying significant variation beyond chance. The associated *p*-value of less than 0.01 confirmed the statistical significance of this heterogeneity. The τ^2^ value of 1.9289 provided insight into the extent to which genuine differences in *Streptococcus suis* prevalence rates contributed to the observed heterogeneity.

Table 3 shows the overall meta-analysis of the prevalence patterns of various zoonotic bacterial pathogens in pig populations in India from 2010 to 2023.

## 4. Discussion

Zoonotic bacterial pathogens within the pig production system represent a significant public health concern due to their potential to transmit diseases to humans. In this study, we performed a systematic meta-analysis of 73 published studies conducted across India, spanning between 2010 to 2023 to assess the prevalence patterns of various zoonotic bacterial pathogens in pigs. The findings have provided some valuable insights into the distribution and prevalence of these pathogens, along with their potential implications for public health and veterinary interventions.

The present analysis revealed distinct patterns of prevalence across different bacterial pathogens, which have zoonotic importance. *Staphylococcus* spp. exhibited the highest estimated prevalence with a random effects proportion of 0.35 (95% CI: [0.21; 0.52]), followed by *Clostridium* spp. with a random effects proportion of 0.23 (95% CI: [0.11; 0.41]). The prevalence of *Staphylococcus* spp. was notably consistent with previous studies, closely aligning with Latha et al. (2017) at 48%, Fahrion et al. (2014) at 47%, Kumar et al. (2014) at 28%, Yaiphathoi et al. (2020) at 26%, and Zehra et al. (2019) at 21% [38,69,75,83,85]. Similarly, the prevalence of *Clostridium* spp. closely corresponded to the findings of previous studies, aligning notably with Das et al. (2017) at 37%, Hussain et al. (2021) at 33%, and Hazarika et al. (2023) at 15% [40,41,44]. In contrast, *Brucella* spp. and *Salmonella* spp. showed lower estimated random effects proportions of 0.06 (95% CI: [0.03; 0.13]) and 0.1 (95% CI: [0.06; 0.16]), respectively. The prevalence of *Brucella* spp. in the present study corroborated the findings of Jindal et al. (2017), Shome et al. (2019), and Fahrion et al. (2014) which showed the prevalence to be ten, eight, and six percent, respectively [28,31,38]. The prevalence of *Salmonella* spp. was consistent with the findings of several prior studies, including Kumar et al. (2014) at 18% and 9%, Chaudhury et al. (2015) at 14%, Chaudhary et al. (2016) at 14%, Kalambhe et al. (2016) at 6%, Lalruatdiki et al. (2018) at 13%, and Kylla et al. (2019) at 8% [47,50,70,71,73,74]. *E. coli* exhibited a moderate estimated prevalence in pig populations with a random effects proportion of 0.24 (95% CI: [0.13; 0.40]). Similarly, *Listeria monocytogenes* and *Streptococcus suis* also demonstrated moderate prevalence levels with random effects proportions of 0.14 (95% CI: [0.08; 0.22]) and 0.13 (95% CI: [0.06; 0.27]), respectively. The prevalence of *E. coli* closely resembled the findings of previous studies, aligning notably with Mandakini et al. (2020) at 32%, Mandakini et al. (2015) at 25%, Tamta et al. (2020) at 25%, Lalruatdiki et al. (2018) at 24%, and Kumar et al. (2021) at 24% and 33% [50,51,54,59,62]. The prevalence of *Listeria monocytogenes* in the present study was consistent with the findings of Suryawanshi et al. (2017) at 16% and 9%, Vaidya et al. (2018) at 20%, and Raorane et al. (2014) at 13% [64,65,67]. In the present study, it was also observed that the prevalence of *Streptococcus suis* was on par with the findings of several researchers [90,94,96].

The study also revealed that heterogeneity was a common feature among the studies, with I^2^ values exceeding 50% for all of the pathogens. This indicated substantial variability among the included studies. Furthermore, funnel plots were used to assess publication bias, and in some cases, asymmetry was observed, suggesting the potential influence of small-study effects or publication bias.

The higher prevalence of zoonotic bacterial pathogens as observed in the present study, such as *Staphylococcus* spp. and *Clostridium* spp. underscores the need for continued surveillance, targeted interventions and control measures both at the farm and processing levels to reduce the risk of zoonotic disease transmission from pigs to humans. Serological and molecular epidemiological studies can help in elucidating the genetic diversity and evolution of these pathogens [99]. It has also been observed that the studies included in the present meta-analysis commonly used techniques like biochemical tests and PCR [44,56,97] followed by ELISA [34,37,64] and lateral flow assays [33] for the detection of bacterial pathogens. It is very much desired that longitudinal studies are needed to monitor the changes in prevalence over time and to assess the effectiveness of control measures.

## 5. Conclusions

The meta-analysis covering 2010 to 2023 revealed a significant prevalence of zoonotic bacterial pathogens among the pig population in India. The study elucidated the prevalence patterns of zoonotic bacterial pathogens in the Indian pig population, with *Staphylococcus* spp. emerging as the most prevalent bacterial pathogen in pigs, closely followed by *E. coli* and *Clostridium* spp., while *Brucella* spp. and *Salmonella* spp. exhibited lower prevalence rates. Additionally, *Listeria monocytogenes* and *Streptococcus suis* demonstrated moderate prevalence among zoonotic bacterial pathogens in the Indian pig population. These findings underscore the urgent need for adopting a One Health approach, which recognizes the interconnectedness of animal and human health to effectively mitigate economic losses and mitigate zoonotic risks.

## Figures and Tables

**Table 1:** Details of inclusion and exclusion criteria used in the study.

Sl. No.	Criteria	Inclusion Criteria	Exclusion Criteria
1	Study design	Observational	Reviews, editorials, commentaries, and non-observational studies (e.g., experimental, or interventional studies
2	Geographical area	Specified to India only	Study radius outside India
3	Publication year	From 2010 to 2023	Studies other than said period (Before 2009 and after 2023)
4	Selection of bacteria	Having zoonotic importance and at least 6 publications within the study range	Non-zoonotic bacteria and less than 6 numbers of publication within the study range
5	Specified for the organisms	*Brucella* spp., *Clostridium* spp., *E. coli*, *Listeria monocytogenes*, *Salmonella* spp., *Staphylococcus* spp., *Streptococcus suis*	Other than mentioned organisms
6	Sample size	More than 2 samples	Less than 2 samples
7	Target animal	Swine	Other than mentioned animal
8	Publication type	Peer-Reviewed	Non-peer-reviewed articles, conference abstracts, or unpublished data
9	Language	English	Non-English language publications
10	Sample source	Blood, tissue, body fluids, stool samples, farm waste and environmental samples etc.	Samples from human and other animals

**Table 2:** List of published research articles and details of studies included in the meta-analysis of zoonotic bacterial pathogens of pigs in India from 2010–2023.

Sl. No.	Author’s Name	Year of Publication	Sample Size	Organism	Number of Positives	Percent Positive	Study Area	References
1	Shome et al., 2019	2019	575	*Brucella*	236	41.04	Southern India	[28]
2	Shome et al., 2019	2019	575	*Brucella*	47	8.17	Southern India	[28]
3	Gogoi et al., 2017	2017	115	*Brucella*	0	0.00	North Eastern India	[29]
4	Kalleshamurthya et al., 2019	2019	1121	*Brucella*	5	0.45	North East India	[30]
5	Jindal et al., 2017	2017	330	*Brucella*	9	2.73	Northern India	[31]
6	Jindal et al., 2017	2017	330	*Brucella*	8	2.42	Northern India	[31]
7	Jindal et al., 2017	2017	330	*Brucella*	10	3.03	Northern India	[31]
8	Jindal et al., 2017	2017	40	*Brucella*	4	10.00	Northern India	[31]
9	Kaur et al., 2020	2020	34	*Brucella*	8	23.53	Northern India	[32]
10	Kaur et al., 2020	2020	90	*Brucella*	15	16.67	Northern India	[32]
11	Kaur et al., 2020	2020	90	*Brucella*	11	12.22	Northern India	[32]
12	Kavya et al., 2017	2017	225	*Brucella*	88	39.11	Southern India	[33]
13	Kavya et al., 2017	2017	225	*Brucella*	74	32.89	Southern India	[33]
14	Tadepalli et al., 2011	2011	1184	*Brucella*	221	18.67	Southern India	[34]
15	Tadepalli et al., 2011	2011	1184	*Brucella*	359	30.32	Southern India	[34]
16	Tadepalli et al., 2011	2011	1184	*Brucella*	356	30.07	Southern India	[34]
17	Shakuntala et al., 2016	2016	2583	*Brucella*	20	0.77	North Eastern India	[35]
18	Shakuntala et al., 2016	2016	2583	*Brucella*	4	0.15	North Eastern India	[35]
19	Shakuntala et al., 2020	2019	3597	*Brucella*	13	0.36	North Eastern India	[36]
20	Shakuntala et al., 2020	2019	3597	*Brucella*	72	2.00	North Eastern India	[36]
21	Shome et al., 2016	2016	2576	*Brucella*	365	14.17	mix	[37]
22	Kavya et al., 2017	2017	225	*Brucella*	70	39.11	Southern India	[33]
23	Fahrion et al., 2014	2014	53	*Brucella*	3	5.66	North Eastern India	[38]
24	Yadav et al., 2018	2018	111	*Clostridium*	4	3.60	Southern India	[39]
25	Das et al., 2017	2017	41	*Clostridium*	15	36.59	North Eastern India	[40]
26	Hazarika et al., 2023	2023	41	*Clostridium*	6	14.63	North Eastern India	[41]
27	Yadav et al., 2017	2017	154	*Clostridium*	59	38.31	Eastern India	[42]
28	Hussain et al., 2016	2016	233	*Clostridium*	29	12.45	North Eastern India	[43]
29	Hussain et al., 2021	2021	116	*Clostridium*	38	32.76	North Eastern India	[44]
30	Hussain et al., 2017	2017	2	*Clostridium*	2	100.00	North Eastern India	[45]
31	Kataria et al., 2014	2014	100	*E. coli*	51	51.00	North Eastern India	[46]
32	Kylla et al., 2019	2019	457	*E. coli*	6	1.31	North Eastern India	[47]
33	Regon et al., 2014	2014	150	*E. coli*	150	100.00	North Eastern India	[48]
34	Tamta et al., 2020	2020	124	*E. coli*	55	44.35	mix	[49]
35	Tamta et al., 2020	2020	21	*E. coli*	9	42.86	Southern India	[49]
36	Lalruatdiki et al., 2018	2018	228	*E. coli*	58	25.44	North Eastern India	[50]
38	Kumar et al., 2021	2021	37	*E. coli*	9	24.32	North Eastern India	[51]
39	Kumar et al., 2021	2021	49	*E. coli*	16	32.65	North Eastern India	[51]
40	Debbarma et al., 2020	2020	420	*E. coli*	66	15.71	North Eastern India	[52]
41	Begum et al., 2013	2013	1260	*E. coli*	65	5.16	North Eastern India	[53]
42	Tamta et al., 2020	2020	71	*E. coli*	35	49.30	Northern India	[54]
43	Tamta et al., 2020	2020	84	*E. coli*	20	23.81	Southern India	[54]
44	Nirupama et al., 2018	2018	741	*E. coli*	243	32.79	mix	[55]
45	Samanta et al., 2015	2015	200	*E. coli*	76	38.00	Eastern India	[56]
46	Puii et al., 2019	2019	164	*E. coli*	6	3.66	North Eastern India	[57]
47	Rajkhowa et al., 2014	2014	782	*E. coli*	113	14.45	North Eastern India	[58]
48	Mandakini et al., 2015	2015	170	*E. coli*	43	25.29	North Eastern India	[59]
49	Kumar et al., 2019	2019	531	*E. coli*	345	64.97	mix	[60]
50	Kylla et al., 2020	2020	1286	*E. coli*	30	2.33	North Eastern India	[61]
51	Kylla et al., 2020	2020	1286	*E. coli*	42	3.27	North Eastern India	[61]
52	Lalruatdiki et al., 2018	2018	867	*E. coli*	221	25.49	North Eastern India	[50]
53	Mandakini et al., 2020	2020	258	*E. coli*	83	32.17	North Eastern India	[62]
54	Mandakini et al., 2020	2020	258	*E. coli*	29	11.24	North Eastern India	[62]
55	Raorane et al., 2015	2015	501	*Listeria*	31	6.19	Western India	[63]
56	Suryawanshi et al., 2017	2017	92	*Listeria*	15	16.30	Western India	[64]
57	Suryawanshi et al., 2017	2017	92	*Listeria*	5	5.43	Western India	[64]
58	Suryawanshi et al., 2017	2017	92	*Listeria*	8	8.70	Western India	[64]
59	Vaidya et al., 2018	2018	50	*Listeria*	10	20.00	Central India	[65]
60	Fahrion et al., 2014	2014	91	*Listeria*	36	39.56	North Eastern India	[38]
61	Sarangi et al., 2012	2012	13	*Listeria*	4	30.77	Eastern India	[66]
62	Raorane et al., 2014	2014	215	*Listeria*	27	12.56	Northern India	[67]
63	Sharma et al., 2013	2013	55	*Salmonella*	16	29.09	Northern India	[68]
64	Kumar et al., 2014	2014	50	*Salmonella*	9	18.00	Southern India	[69]
65	Kumar et al., 2014	2014	93	*Salmonella*	8	8.60	Northern India	[70]
66	Chaudhary et al., 2015	2015	270	*Salmonella*	37	13.70	Western India	[71]
67	Kylla et al., 2016	2016	20	*Salmonella*	5	25.00	North Eastern India	[72]
68	Chaudhary et al., 2016	2016	270	*Salmonella*	37	13.70	Western India	[73]
69	Kalambhe et al., 2016	2016	100	*Salmonella*	6	6.00	Western India	[74]
70	Latha et al., 2017	2017	310	*Salmonella*	0	0.00	Southern India	[75]
71	Das et al., 2018	2018	200	*Salmonella*	5	2.50	North Eastern India	[76]
72	Lalruatdiki et al., 2018	2018	228	*Salmonella*	30	13.16	North Eastern India	[50]
73	Chakraborty et al., 2019	2019	50	*Salmonella*	9	18.00	North Eastern India	[77]
74	Mahindroo1 et al., 2019	2019	208	*Salmonella*	52	25.00	Northern India	[78]
75	Kylla et al., 2019	2019	457	*Salmonella*	38	8.32	Northern India	[79]
76	Borah et al., 2022	2022	1231	*Salmonella*	88	7.15	North Eastern India	[80]
77	Kumar et al., 2014	2014	50	*Staphylococcus*	14	28.00	Southern India	[69]
78	Fahrion et al., 2014	2014	19	*Staphylococcus*	9	47.37	North Eastern India	[38]
79	Zehra et al., 2017	2017	28	*Staphylococcus*	20	71.43	Northern India	[81]
80	Rajkhowa et al., 2016	2016	698	*Staphylococcus*	49	7.02	North Eastern India	[82]
82	Yaiphathoi et al., 2020	2020	50	*Staphylococcus*	13	26.00	North Eastern India	[83]
83	Latha et al., 2017	2017	310	*Staphylococcus*	149	48.06	Southern India	[75]
84	Kalai et al., 2020	2020	60	*Staphylococcus*	44	73.33	North Eastern India	[84]
85	Zehra et al., 2019	2019	131	*Staphylococcus*	27	20.61	Northern India	[85]
86	Yaiphathoi et al., 2019	2019	50	*Staphylococcus*	13	26.00	North Eastern India	[86]
88	Savariraj et al., 2018	2018	120	*Staphylococcus*	82	68.33	Southern India	[87]
89	Baruah et al., 2016	2016	349	*Staphylococcus*	34	9.74	North Eastern India	[88]
90	Devi et al., 2017	2017	497	*Streptococcus*	7	1.41	North Eastern India	[89]
91	Anand et al., 2016	2016	100	*Streptococcus*	9	9.00	Northern India	[90]
92	Dinesh et al., 2020	2020	243	*Streptococcus*	14	5.76	Northern India	[91]
93	Dinesh et al., 2022	2022	664	*Streptococcus*	41	6.17	Northern and North Eastern India	[92]
94	Pegu et al., 2020	2020	116	*Streptococcus*	32	27.59	North Eastern India	[93]
95	Sonowal et al., 2014	2014	126	*Streptococcus*	15	11.90	North Eastern India	[94]
96	Rajkhowa et al., 2021	2021	365	*Streptococcus*	62	16.99	North Eastern India	[95]
97	Rajkhowa et al., 2017	2017	34	*Streptococcus*	27	79.41	North Eastern India	[96]
98	Devi et al., 2017	2017	497	*Streptococcus*	35	7.04	North Eastern India	[97]
99	Vishva et al., 2022	2022	563	*Streptococcus*	184	32.68	Northern India	[98]

**Table 3:** Meta-analysis of the prevalence patterns of various zoonotic bacterial pathogens in pig populations in India from 2010 to 2023.

Organism	Total Events	Common Effect		Random Effects		Heterogeneity (I^2^)	Variance (τ^2^)	*p*-Value
		Proportion	95% CI (Common Effect)	Proportion	95% CI (Random Effects)			
*Brucella* spp.	23,846	0.09	[0.08; 0.09]	0.06	[0.03; 0.13]	99%	3.4092	0
*Clostridium* spp.	698	0.22	[0.19; 0.25]	0.23	[0.11; 0.41]	90%	1.0815	<0.01
*E. coli*	9544	0.19	[0.18; 0.19]	0.24	[0.13; 0.40]	98%	3.1956	<0.01
*Listeria monocytogenes*	1146	0.12	[0.10; 0.14]	0.14	[0.08; 0.22]	91%	0.5654	<0.01
*Salmonella* spp.	3542	0.1	[0.09; 0.11]	0.1	[0.06; 0.16]	87%	1.1165	<0.01
*Staphylococcus* spp.	1865	0.24	[0.22; 0.26]	0.35	[0.21; 0.52]	98%	1.3396	<0.01
*Streptococcus suis*	3205	0.13	[0.12; 0.15]	0.13	[0.06; 0.27]	97%	1.9289	<0.01

## Data Availability

The datasets used during the study will be available upon request to the corresponding author.
